# Diabetic ketoacidosis induced by liraglutide overdose for weight loss in a type 2 diabetes patient: A case report

**DOI:** 10.1097/MD.0000000000046197

**Published:** 2026-01-23

**Authors:** Hekai Xu, Yuqin Zhang

**Affiliations:** a Department of Cardiovascular Medicine, Center of Coronary Circulation, Xiangya Hospital of Central South University, Changsha, China; b Department of Geriatric Medicine, Center of Coronary Circulation, Xiangya Hospital of Central South University, Changsha, China; c Department of General Medicine, Xiangya Hospital of Central South University, Changsha, China; d State Key Laboratory of Advanced Medical Materials and Devices, Tianjin Key Laboratory of Biomedical Materials, Key Laboratory of Biomaterials and Nanotechnology for Cancer Immunotherapy, Institute of Biomedical Engineering, Chinese Academy of Medical Sciences and Peking Union Medical College, Tianjin, PR China.

**Keywords:** diabetic ketoacidosis, GLP-1 receptor agonist, glycemic control, liraglutide, overdose, type 2 diabetes mellitus

## Abstract

**Background:**

Type 2 diabetes mellitus is a major global health concern. Liraglutide, a glucagon-like peptide-1 receptor agonist, is widely used for glycemic control and weight loss. While generally safe, its misuse, particularly overdose, poses severe risks that are poorly documented. This case report aims to describe the clinical course and management of a life-threatening diabetic ketoacidosis (DKA) induced by a massive liraglutide overdose, highlighting the critical importance of patient education and adherence to prescribing guidelines.

**Methods:**

We present a case report of a 43-year-old female with type 2 diabetes mellitus who self-administered liraglutide at 10 times the recommended dose (cumulative 18 mg over 3 days).

**Results:**

Following the overdose, the patient was diagnosed with severe DKA (pH 6.9), with markedly elevated β-hydroxybutyrate and hyperkalemia. Conventional management failed to correct the life-threatening acidosis, necessitating salvage hemodialysis, which rapidly stabilized her metabolic parameters.

**Conclusion:**

This case underscores the dangers of liraglutide overdose, particularly for weight loss purposes, and highlights the critical need for strict adherence to prescribed dosages and comprehensive patient education. Clinicians should vigilantly monitor liraglutide therapy to prevent severe complications like DKA. In refractory DKA with profound acidosis (pH ≤ 7.0) or end-organ compromise, hemodialysis may be considered for rapid detoxification and stabilization. This report serves as a critical reminder of the potential dangers associated with glucagon-like peptide-1 receptor agonist misuse.

## 1. Introduction

Type 2 diabetes mellitus (T2DM) is a significant global health challenge and a major contributor to morbidity and mortality worldwide.^[1]^ Liraglutide (Victoza, Novo Nordisk A/S, Bagsværd, Denmark), a predominant glucagon-like peptide-1 (GLP-1) receptor agonist in clinical use, has demonstrated high levels of glycemic benefit in head-to-head studies versus other GLP-1 receptor agonists,^[2]^ along with several other clinical benefits, including reductions in body weight and systolic blood pressure and low rates of hypoglycemia.^[3]^ Due to its ability to induce weight loss, it is favored by women in China.

## 2. Case presentation

This case report was approved by the Institutional Review Board of Xiangya Hospital of Central South University. Written informed consent was obtained from the patient for publication of her clinical details.

**Figure 1.**
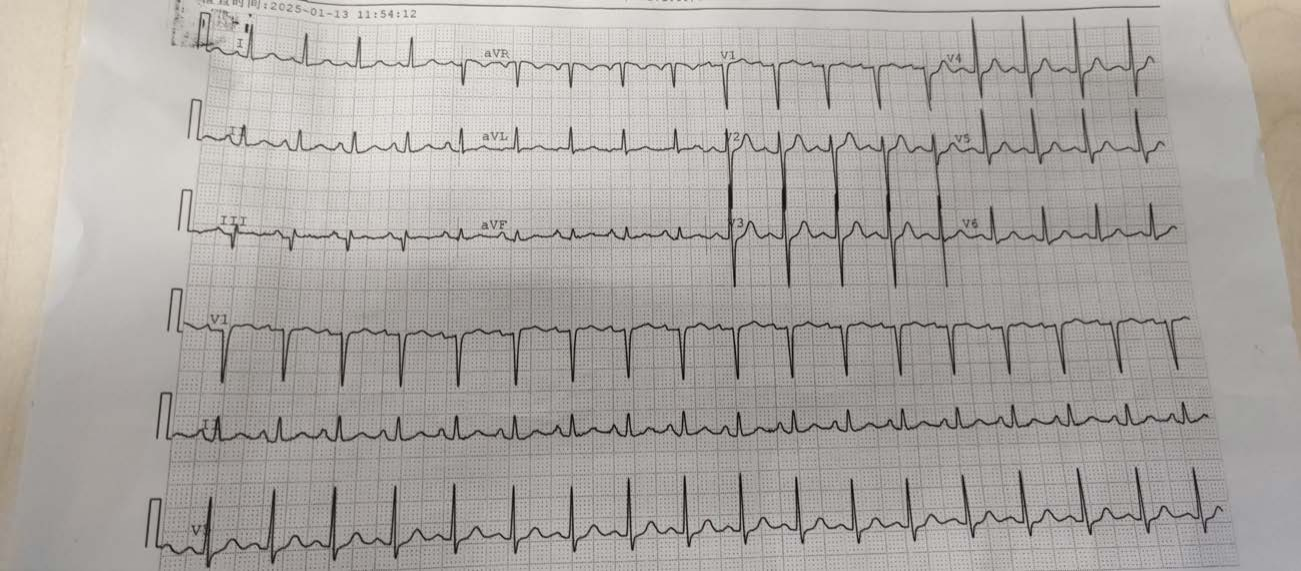
Electrocardiogram report of the patient before treatment.

A 43-year-old female with a known history of type 2 diabetes mellitus, previously managed with insulin therapy (insulin regimen: short-acting insulin 12 units 3 times daily), presented to the emergency department with a 3-day history of palpitations, chest pain, and progressive shortness of breath, which acutely worsened over the past 2 hours. On physical examination, the patient appeared distressed and in moderate respiratory distress. Vital signs were as follows: heart rate 128 bpm, blood pressure 129/66 mm Hg, respiratory rate 24 breaths per minute, and oxygen saturation 96%. Auscultation revealed bilateral basal crackles in the lungs, and the cardiac examination showed a tachycardic but regular rhythm. The abdomen was soft, non-tender, and no hepatomegaly or splenomegaly was noted. The patient was diagnosed with T2DM 5 years prior, insulin therapy was initiated 8 months before admission, without concomitant use of metformin, SGLT2 inhibitors, or other antihyperglycemic agents. Three days prior to admission, motivated by a desire for rapid weight loss, the patient had self-administered liraglutide subcutaneously at a dosage of 3 times daily, totaling approximately 18 mg over 3 days. She experienced abdominal bloating, decreased appetite, palpitations, and chest discomfort following the initiation of liraglutide. On admission, she was found that heart rate was 171 beats per minute (Fig. 1) and respiratory rate was 23 breaths per minute. Laboratory examination was notable for a potassium: 5.32 mmol/L (reference range: 3.5–5.5 mmol/L), β-hydroxybutyrate: 3.2 mmol/L (reference range: 0.0–0.3 mmol/L), with profound acidemia on venous blood gas with pH 6.9 (reference range 7.33–7.43), pCO_2_ 123 mm Hg (reference range: 38–50 mm Hg), HCO_3_ < 5 mEq/L (reference range 20–30 mEq/L), and creatinine 101 μmol/L (reference range: 59–104 μmol/L, her renal function was within normal limits prior to this incident). Her hemoglobin hemoglobin A1C was 11.4% (reference range: <5.6%) and her random blood glucose was 15.99 mmol/L (reference range: <7.8 mmol/L) (Table 1). Pancreatitis was ruled out based on the absence of abdominal pain and normal pancreatic enzymes (amylase 28.6 U/L; lipase within normal limits). A diagnosis of diabetic ketoacidosis (DKA) was made and immediate initiation of the DKA management protocol was undertaken, including aggressive fluid resuscitation, intravenous insulin infusion for glycemic control and ketone clearance. Hemodialysis was initiated due to profound metabolic acidosis (pH 6.9) refractory to intravenous bicarbonate therapy, along with hyperkalemia and evolving acute kidney injury, consistent with current recommendations for extracorporeal therapy in severe DKA. Following hemodialysis, the patient showed significant clinical improvement with resolution of dyspnea and chest tightness. On hospital day 3, she was transferred to a local tertiary center for ongoing care (Table 2).

**Table 1:** Laboratory findings on admission.

Parameter	Value on admission	Reference range
Glucose (random)	15.99 mmol/L	<7.8 mmol/L
pH (venous)	6.9	7.33–7.43
pCO_2_ (venous)	123 mm Hg	38–50 mm Hg
HCO₃^‐^	<5 mEq/L	20–30 mEq/L
β-Hydroxybutyrate (β-HB)	3.2 mmol/L	0.0–0.3 mmol/L
Creatinine (Cr)	101 μmol/L	59–104 μmol/L

**Table 2:** Timeline of key events, clinical findings, and interventions.

Date and time (approx.)	Event/intervention	Clinical presentation & key findings
Day 1–3		Progressive shortness of breath and chest pain.
Day 4, 7:30		Progressive shortness of breath and chest pain.
Day 4, 9:30	Emergency department admission	HR 128 bpm, RR 24/min, O_2_ sat 96%.
	Initial lab results	Glucose: 15.99 mmol/L; pH: 6.90; HCO₃^‐^: <5 mEq/L; β-HB: 6.2 mmol/L; K^+^: 5.32 mmol/L; Cr: 101 μmol/L.
Day 4, 15:30	DKA protocol initiated	Correct acid, replenish fluid, lower blood sugar, eliminate ketosis, and supplement potassium
Day 5, 16:21	Hemodialysis initiated	Due to refractory acidosis (pH ≤ 7.0) despite conventional management.
	Lab results	Glucose: 8.39 mmol/L; pH: 7.36; HCO_3_^‐^: 18 mEq/L; β-HB: 2.2 mmol/L; K^+^: 3.64 mmol/L; Cr: 92 μmol/L.
Day 5	Post-dialysis	Significant clinical improvement. Resolution of dyspnea and chest tightness.
	Transfer	Transferred to a tertiary center for ongoing management.

DKA = diabetic ketoacidosis.

## 3. Discussion

Liraglutide, a GLP-1 receptor agonist, is approved for managing T2DM and obesity. It exerts its effects by enhancing glucose-dependent insulin secretion, suppressing glucagon release, delaying gastric emptying, and promoting satiety.^[2,3]^ The standard maximum daily dose for diabetes management is 1.8 mg. However, our case presents a severe complication following a massive overdose.

This report adds to a small but growing number of cases linking glucagon-like peptide-1 receptor agonists (GLP-1RAs) to DKA.^[4,5]^ While most reported cases occur in the context of appropriate dosing alongside insulin discontinuation or other precipitating factors, instances stemming from intentional overdose, as presented here, remain exceedingly rare. Recent evidence suggests that GLP-1RAs may contribute to DKA through several pathophysiological pathways, particularly in high-risk individuals with compromised beta-cell function. Key mechanisms include: dependent insulin secretion: GLP-1RAs require residual β-cell function to stimulate insulin release. In patients with advanced β-cell failure (e.g., misdiagnosed latent autoimmune diabetes in adults or long-standing T2DM), they fail to compensate for insulin deficiency, accelerating lipolysis and ketogenesis.^[4,6]^ Catabolic state induction: pronounced appetite suppression and gastrointestinal adverse effects (nausea, vomiting) significantly reduce caloric and fluid intake. This creates a catabolic state that promotes free fatty acid release and hepatic ketone production, exacerbated by dehydration.^[4,5]^ Sodium-hydrogen exchanger 3 (NHE3) inhibition: experimental data indicate that GLP-1 signaling reduces renal bicarbonate reabsorption via suppression of the NHE3, potentially worsening metabolic acidosis.^[6]^

In our case, the patient’s preexisting T2DM with poor glycemic control (hemoglobin A1c 11.4%) indicated significantly impaired insulin reserve. The massive liraglutide overdose (18 mg over 3 days) precipitated a perfect storm: its potent appetite-suppressant effects led to severe caloric restriction and dehydration, while its mechanism of action was insufficient to compensate for her underlying insulin deficiency. This aligns with the established paradigm where GLP-1RA monotherapy without adequate endogenous insulin reserve is a critical risk factor for DKA. The decision to initiate hemodialysis in this case was a critical intervention for life-saving purposes. It was primarily driven by the presence of profound, refractory metabolic acidosis (pH ≤ 6.9) that was unresponsive to conventional management with aggressive intravenous fluids and insulin therapy. At such a severely low pH, the risk of severe myocardial dysfunction, circulatory collapse, and fatal arrhythmias is exceedingly high. Hemodialysis serves as an effective extracorporeal therapy to rapidly correct acidemia by clearing keto-anions and hydrogen ions, and simultaneously managing associated electrolyte imbalances, such as hyperkalemia. This approach aligns with emerging literature supporting the role of renal replacement therapy as a salvage maneuver in cases of severe, complicated DKA where rapid correction is necessary to prevent end-organ damage and mortality.^[7,8]^

This case underscores several critical lessons for clinical practice. First, it highlights the paramount importance of patient education. Patients prescribed GLP-1RAs must be rigorously instructed on: strict adherence to prescribed dosing limits; the grave risks of arbitrarily discontinuing insulin therapy without medical supervision; and early recognition of ketosis symptoms (e.g., nausea, vomiting, abdominal pain, excessive thirst, shortness of breath). Proactive monitoring of blood glucose and ketone levels is essential, especially during therapy initiation or in patients with a history of poor glycemic control.

Finally, this incident reflects broader public health concerns. The rising popularity of GLP-1RAs for weight loss, fueled by social media and off-label use, increases the risk of misuse among individuals desperate for rapid results. Clinicians must therefore exercise heightened vigilance when prescribing these agents, particularly for obesity, carefully screening for inappropriate motivation or history of disordered eating. A concerted effort involving healthcare providers, regulatory bodies, and public health messaging is needed to promote safe prescribing practices and manage patient expectations, thereby mitigating the risks of severe complications such as DKA.

## 4. Limitations

This study has limitations inherent to its design as a single case report. The findings and management strategies described are specific to this individual’s unique clinical scenario and may not be generalizable to all patients. Furthermore, the exact contribution of each pathophysiological mechanism (e.g., NHE3 inhibition) to the severe acidosis remains theoretical and is based on emerging preclinical evidence.

## Author contributions

**Writing – original draft, review & editing:** Hekai Xu, Yuqin Zhang.
